# Cystathionine β-synthase TtCbs1 from *Tetrahymena thermophila* catalyzes the synthesis of CdS quantum dots for methyl orange decolorization

**DOI:** 10.1128/aem.01255-25

**Published:** 2025-09-24

**Authors:** Wenliang Lei, Juan Liu, Jing Xu, Wei Wang

**Affiliations:** 1 Key Laboratory of Chemical Biology and Molecular Engineering of Ministry of Education, Institute of Biotechnology, Shanxi University 547807, Taiyuan, China; 2 School of Life Science, Shanxi University 630971, Taiyuan, China; 3 Shanxi Key Laboratory of Biotechnology, Taiyuan, China; Kyoto University, Kyoto, Japan

**Keywords:** cystathionine β-synthase, cysteine, cadmium sulfide quantum dots, photocatalytic activity, *Tetrahymena thermophila*

## Abstract

Cystathionine β-synthase (CBS) is a pivotal enzyme in the transsulfuration metabolic pathway, catalyzing the synthesis of H_2_S and cystathionine. These metabolites mediate stress responses and maintain cellular redox homeostasis. In *Tetrahymena thermophila*, *TtCBS1* expression was upregulated under cadmium stress, with cysteine further enhancing its transcription. The recombinant His-TtCbs1 protein was expressed in *Escherichia coli* and purified by affinity chromatography. The His-TtCbs1 catalyzed the synthesis of monodisperse cadmium sulfide quantum dots (CdS QDs) in cysteine and Cd^2+^ solutions. The QDs exhibited an average diameter of 3.51 nm, with size increasing over reaction time. Fourier-transform infrared spectroscopy revealed characteristic amide I (1,640 cm⁻¹) and amide II (1,520 cm⁻¹) peaks, confirming interactions of CdS and the protein. Cysteine and glutathione stabilized the CdS QDs in the solution. Under UV light irradiation, the CdS QDs decolorized 91% of methyl orange. This study shows that CBS from protists mitigates cadmium toxicity by synthesizing CdS QDs. The biosynthesized CdS QDs also function as effective biocatalysts for the decolorization of organic dyes.

**IMPORTANCE:**

The significant upregulation of *TtCBS*1 in *Tetrahymena thermophila* under cadmium stress indicates that this enzyme is a key player in the organism's defense mechanism against cadmium toxicity. Cadmium sulfide (CdS) nanoparticles were synthesized using the TtCbs1 single-enzyme system *in vitro*. Cysteine and glutathione play a critical role in controlling the growth of biosynthetic CdS particle size. Understanding the precise mechanisms by which cysteine and glutathione control particle size could lead to the development of more precise and efficient biomineralization processes. The synthesized CdS quantum dots exhibited significant photocatalytic activity. This work highlights the potential of cystathionine β-synthase from protists in metal detoxification and environmental remediation.

## INTRODUCTION

Biomineralization represents a critical physiological process across organisms (1, 2). Microorganisms have evolved diverse mineralization strategies to thrive in varied ecological niches (3, 4). The simulation of biomineralization using living cells and their secretions to synthesize nanomaterials has emerged as a crucial strategy for green nanofabrication (5, 6). Cadmium sulfide (CdS), a prominent semiconductor with exceptional optoelectronic properties, has garnered significant attention for photocatalytic applications, particularly in the degradation of organic pollutants (7–9). Conventional CdS synthesis relies on high-temperature, high-pressure reactions between sulfur and cadmium precursors, posing environmental risks due to toxic byproducts and energy-intensive processes (10–12). In contrast, biological CdS synthesis—harnessing microbial metabolic pathways—offers a sustainable, eco-friendly alternative with substantial industrial potential.

Hydrogen sulfide (H_₂_S) is a critical mediator in CdS biomineralization, with organisms employing diverse metabolic pathways for its production (13, 14). In mammals, cystathionine β-synthase (CBS) and cystathionine γ-lyase (CSE) generate H_2_S from homocysteine, while 3-mercaptopyruvate sulfurtransferase (3MST) utilizes 3-mercaptopyruvate as a substrate (15–17). Bacteria further contribute via sulfite reductases, which reduce SO_3_²⁻ to H_2_S (18, 19). Under cadmium stress, the deep-sea bacterium *Idiomarina* sp. OT37-5b produces CdS, with cysteine supplementation enhancing cellular CdS synthesis (20). *Escherichia coli* generates CdS quantum dots (CdS QDs) under cadmium stress (21). The threonine dehydrogenase psTD of deep-sea bacterium *Pseudomonas stutzeri* 273 catalyzes *in vitro* CdS QDs synthesis (22). The CSE from the *Stenotrophomonas maltophilia* produces H_₂_S from cysteine, yielding monodisperse CdS QDs upon Cd²^+^ exposure (23).

Protists, with their rich species diversity and extensive genetic resources, offer immense value for both ecological and biotechnological research. *Tetrahymena thermophila*, a commonly used freshwater protist in toxicological research, is highly sensitive to the toxicity of pesticides, heavy metals, and nanomaterials. This sensitivity, combined with its rapid reproduction rate and ease of cultivation, makes it an ideal model organism for investigating responses to metal stress and other environmental contaminants (24–28). Our previous studies identified cysteine synthase (TtCsa1) as a key H_₂_S producer from cysteine in this organism (29–31). Beyond enhancing cadmium tolerance, TtCsa1-mediated *in vitro* CdS biomineralization yielded photocatalytic nanomaterials capable of degrading methylene blue under UV light (32). To further exploit single-enzyme CdS synthesis, we investigated *TtCBS1*—a transsulfuration pathway enzyme—in *T. thermophila* under cadmium stress. His-tagged TtCbs1 was heterologously expressed in *E. coli*, purified, and utilized to catalyze monodisperse CdS QD formation *in vitro*. The resulting biosynthesized QDs demonstrated rapid methyl orange decolorization under UV irradiation. This work advances the use of protozoan enzymes in sustainable nanomaterial synthesis and highlights their potential for pollutant remediation.

## MATERIALS AND METHODS

### Strains and reagents

The pET28a-sumo-*TtCBS1* plasmid was previously constructed in our laboratory (29). *T. thermophila* B2086 (mating type II) was obtained from the *Tetrahymena* Stock Center at Cornell University (http://tetrahymena.vet.cornell.edu/) and cultured at 30°C in SPP medium (1% peptone, 0.1% yeast extract, 0.2% glucose, and 0.003% sequestrene, pH 7.4). l-cysteine (Shanghai Sangon), cadmium chloride (Beijing Solarbio Technology), reduced glutathione (Shanghai Sangon), and sodium sulfide (Aladdin) were all prepared as stock solutions using ultrapure water.

### RNA extraction and qRT‐PCR

Log-phase *T. thermophila* cells were exposed to Cd²^+^ stress (0, 12.5, 25, 50, 100, and 200 µM) for 12 h at 30°C. The cells were harvested by centrifugation at 3,500 rpm for 4 min and washed with 10 mM Tris-HCl. Total RNA was extracted using RNAiso Plus (Takara). Reverse transcription was performed to synthesize cDNA using a PrimeScript RT reagent Kit (Takara).

The real-time quantitative PCR reaction system was prepared using Hieff qPCR SYBR Green Master Mix (Yeasen Biotech). The qRT-PCR program consisted of an initial denaturation step at 95°C for 30 s, followed by 40 cycles of denaturation at 95°C for 5 s and extension at 60°C for 30 s. The relative expression was calculated via 2^−ΔΔC*t*^ method. 17S rRNA served as the internal reference (33, 34). All qRT-PCR experiments were conducted with three biological replicates and three technical replicates.

### Purification of His-TtCbs1 and biosynthesis of CdS QDs *in vitro*

The pET28a-sumo-*TtCBS1* plasmid was transformed into *E. coli* BL21/DE3. The recombinant strain was cultured overnight in 5 mL LB medium (10 g/L peptone, 10 g/L NaCl, and 5 g/L yeast extract, pH 7.4) at 37°C. Subsequently, the overnight culture was transferred to 500 mL LB medium and incubated at 37°C until the optical density at 600 nm (OD_600_) reached approximately 0.5. Isopropyl β-d-1-thiogalactopyranoside was then added to a final concentration of 0.05 mM to induce recombinant protein expression at 16°C. The *E. coli* cells were harvested and disrupted by ultrasonication. The target protein was purified using affinity chromatography followed by gel filtration chromatography (29).

In the Tris-HCl buffer system (50 mM, pH 8.5), cysteine synthase (0.1 mg/mL), cysteine (4 mM), and cadmium ions (0.5 mM) were added and incubated at 37°C. The UV-visible absorption spectrum of the CdS QDs in the reaction mixture was analyzed using a UV-Vis spectrophotometer. The fluorescence emission spectrum of the CdS QDs was measured using a fluorescence spectrophotometer with a 5 nm excitation slit width at an excitation wavelength of 350 nm (23). The photoluminescence of the reaction system was observed under a UV lamp box with a 365 nm UV light.

After 75 min of reaction, the CdS was precipitated using an equal volume of absolute ethanol. The mixture was then centrifuged at 5,000 rpm for 5 min to collect the precipitate. The precipitate was air-dried and weighed to calculate the yield of CdS. The obtained CdS was dissolved in Tris-HCl buffer containing 4 mM cysteine and stored at low temperature in the dark for further use (35).

### Transmission electron microscopy (TEM)

CdS QDs obtained after a 75-min reaction were immediately mixed with an equal volume of pre-cooled 20 mM glutathione at 4°C. The mixture was then centrifuged in a 3 kDa concentrator to reduce the volume and concentrated. The resulting suspension was ultrasonically dispersed and dropped onto a copper grid, air-dried, and observed using a transmission electron microscope (Tecnai F30, FEI, USA). This technique was used to analyze the size, composition, and crystallography of the nanoparticles. X-ray energy dispersive spectroscopy (XEDS) analysis was conducted at an accelerating voltage of 15 keV for a duration of 100 s to determine the elemental composition of the CdS QDs.

### X-ray diffraction (XRD)

For XRD analysis, CdS precipitates from a 24 h reaction were centrifuged at 8,000 rpm for 30 min, washed two times with anhydrous ethanol, and dried in an oven at 37°C for 48 h. The collected precipitate was ground into a powder and analyzed using an X-ray diffractometer (Panalytical Empyrean, PANalytical B.V., the Netherlands) to obtain the XRD patterns, which provided information on the crystalline structure of the nanoparticles.

### Scanning electron microscopy (SEM)

Bulk CdS particles were gold-coated using an ion sputter coater (ETD2000, Vision Precision Instruments, China) for 5 min to enhance conductivity. The coated samples were examined using a scanning electron microscope (EM-30AX+, COXEM, Korea) at an accelerating voltage of 15 kV to observe the surface morphology of the particles. Energy dispersive X-ray spectroscopy (EDX) analysis was performed at an accelerating voltage of 15 kV for a duration of 100 s to confirm the elemental composition of the CdS particles.

### Particle size control of CdS nanoparticles

To demonstrate the ability of enzymes and cysteine to control the particle size of CdS nanoparticles, three sets of experiments were conducted: single enzyme method: the method involving a single enzyme for CdS synthesis was performed as previously described (32); Na_₂_S with cysteine as capping agent: cysteine (1 mM) and cadmium chloride (0.5 mM) were added to a Tris-HCl buffer (50 mM, pH 8.5), followed by Na_2_S (1 mM), and the reaction was performed at 37°C; Na_₂_S Only: cadmium chloride (0.5 mM) was added to a Tris-HCl buffer (50 mM, pH 8.5), followed by Na_2_S (1 mM), under the same reaction conditions. The optical properties of CdS nanoparticles in solution were characterized using absorbance and fluorescence spectroscopy, along with direct observation under UV light. The CdS produced from each method was ground into a powder and analyzed using a Fourier-transform infrared (FTIR) spectrometer (VERTEX 80v, Bruker, Germany) to obtain the infrared spectra (23).

### Photocatalytic decolorization of methyl orange by CdS QDs

CdS QDs were added to 10 mL of solution, with the reactor positioned 10 cm away from a 100 W ultraviolet lamp (365 nm, 160 mW/cm^2^). A magnetic stirrer was placed beneath the reactor to continuously stir the solution, while a water-cooling system was employed to maintain thermal equilibrium during the reaction. Initially, a stock solution of methyl orange was added to the mixture to achieve specific concentrations ranging from 10 to 70 mg/L. The reaction system was stirred in the dark at room temperature for 1 h to ensure that the adsorption equilibrium was reached (36).

Subsequently, the photocatalytic reaction was initiated at room temperature. Samples were collected at regular intervals, and any insoluble precipitates were removed by centrifugation. The absorbance at 496 nm was measured using a visible light spectrophotometer to calculate the decolorization rate (37). The effects of catalyst dosage, initial dye concentration, and pH on the photocatalytic reaction were systematically investigated. The initial pH of the reaction solution was adjusted using 0.1 M NaOH and 0.1 M H_2_SO_4_. The ultraviolet-visible absorption spectra of methyl orange were measured using a UV-Vis spectrophotometer (38). The decolorization rate was calculated according to Model 1. For lower dye concentrations, the experimental data were fitted to a first-order kinetic model as described in Model 2 to observe the kinetics of the photocatalytic reaction. This approach allows for a comprehensive assessment of the performance of CdS as a photocatalyst and the influencing factors in the decolorization of methyl orange.


(1) $$D\%=(1-(C_{t}-C_{0})/C_{0})\times 100\%$$

(2) $$\ln\left(C_{t}/C_{0}\right)=-k_{app}t$$

where *C*_0_ is the initial concentration of methyl orange, *C_t_* is the concentration of methyl orange at time (*t*, h), *k*_app_ is the apparent pseudo-first-order reaction rate constant (h^−1^), and *t* is the reaction time (h) (39).

### Statistical analysis

The analysis was conducted using IBM SPSS Statistics 27 to perform a one-way analysis of variance (ANOVA). Post hoc comparisons were carried out using Tukey’s HSD (Honestly Significant Difference) test to evaluate whether there were significant differences between groups. Statistical significance is denoted by a single asterisk for *P* < 0.05 and a triple asterisk for *P* < 0.01.

## RESULTS

### Upregulation of *TtCBS1* expression in response to cadmium and cysteine

**Fig 1:**
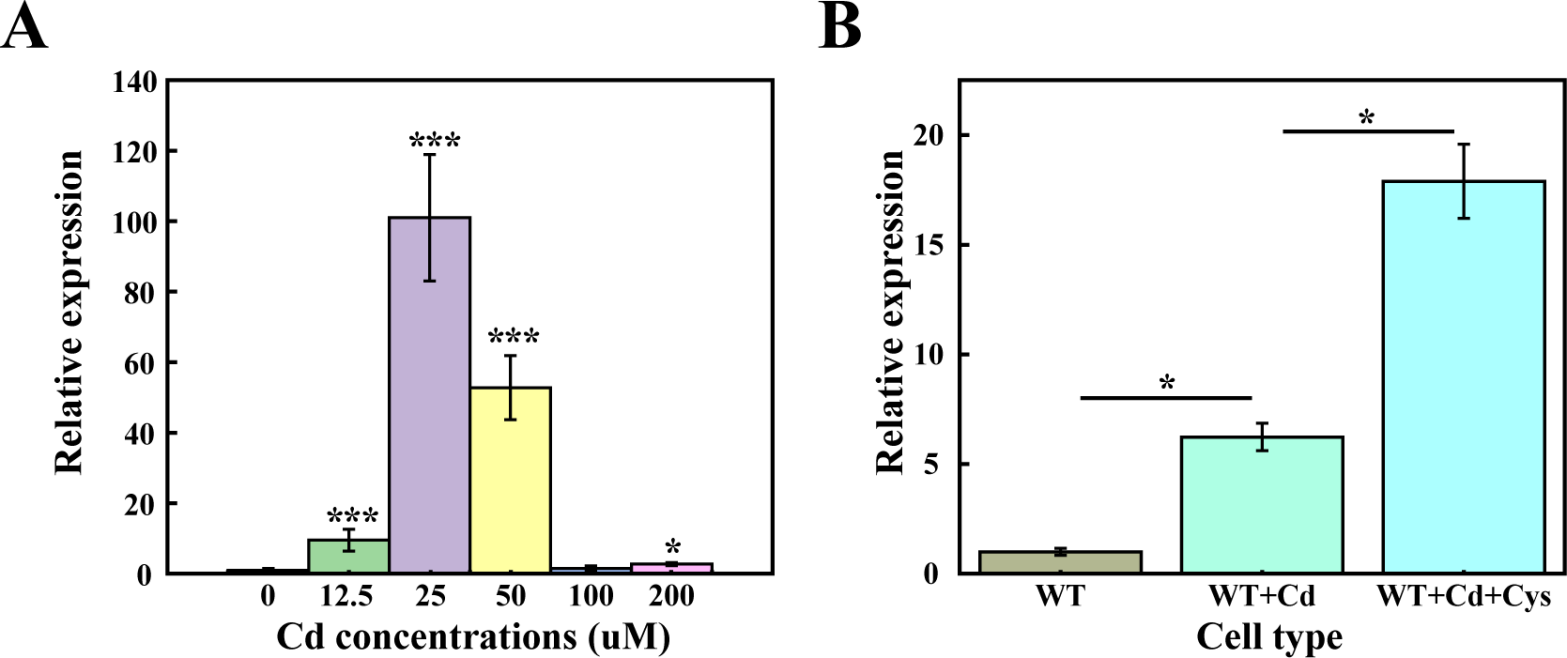
Transcriptional levels of *TtCBS1* under cadmium stress and cysteine supplementation in *T. thermophila*. (**A**) Transcriptional levels of *TtCBS1* under various Cd^2+^ concentrations. *T. thermophila* cells were exposed to varying concentrations of Cd^2+^ (2.5, 25, 50, 100, and 200 µM). Untreated cells (no Cd^2+^) served as a control. Total RNA was extracted, and qRT-PCR was performed to assess *TtCBS1* expression levels. Low Cd^2+^ concentration (12.5 µM): *TtCBS1* expression was significantly upregulated compared to the control; optimal Cd^2+^ concentration (25 µM): *TtCBS1* expression peaked, showing highly significant upregulation; higher Cd^2+^ concentrations (50, 100, and 200 µM): *TtCBS1* expression declined, indicating a possible inhibitory effect due to oxidative stress. (**B**) Cells were exposed to 12.5 µM Cd^2+^ stress. 2 mM cysteine was added to the culture, and *TtCBS1* expression levels were assessed. The addition of cysteine further enhanced *TtCBS1* expression, indicating a synergistic effect between cysteine availability and Cd^2+^ stress. * and *** indicate *P* < 0.05 and *P* < 0.01, respectively.

The transsulfuration pathway interconverts the sulfur-containing amino acids cysteine and homocysteine via the intermediate cystathionine. The transsulfuration pathway in *T. thermophila* operates bidirectionally (29, 40). *TtCSA1* plays a crucial role in the mineralization process of CdS (32). The knockout of *TtCBS1* results in decreased levels of glutathione and cysteine in *Tetrahymena* (29). Basal expression of *TtCBS1* is low but upregulated under cadmium stress, higher Cd concentrations (>50 µM) suppress *TtCBS1* expression, likely due to transcriptional repression from oxidative stress (Fig. 1A). However, cysteine supplementation further enhanced *TtCBS1* expression, suggesting a feedback loop where substrate availability amplifies transsulfuration pathway activity (Fig. 1B).

### His-TtCbs1 catalyzes the biosynthesis of CdS nanoparticles *in vitro*

**Fig 2:**
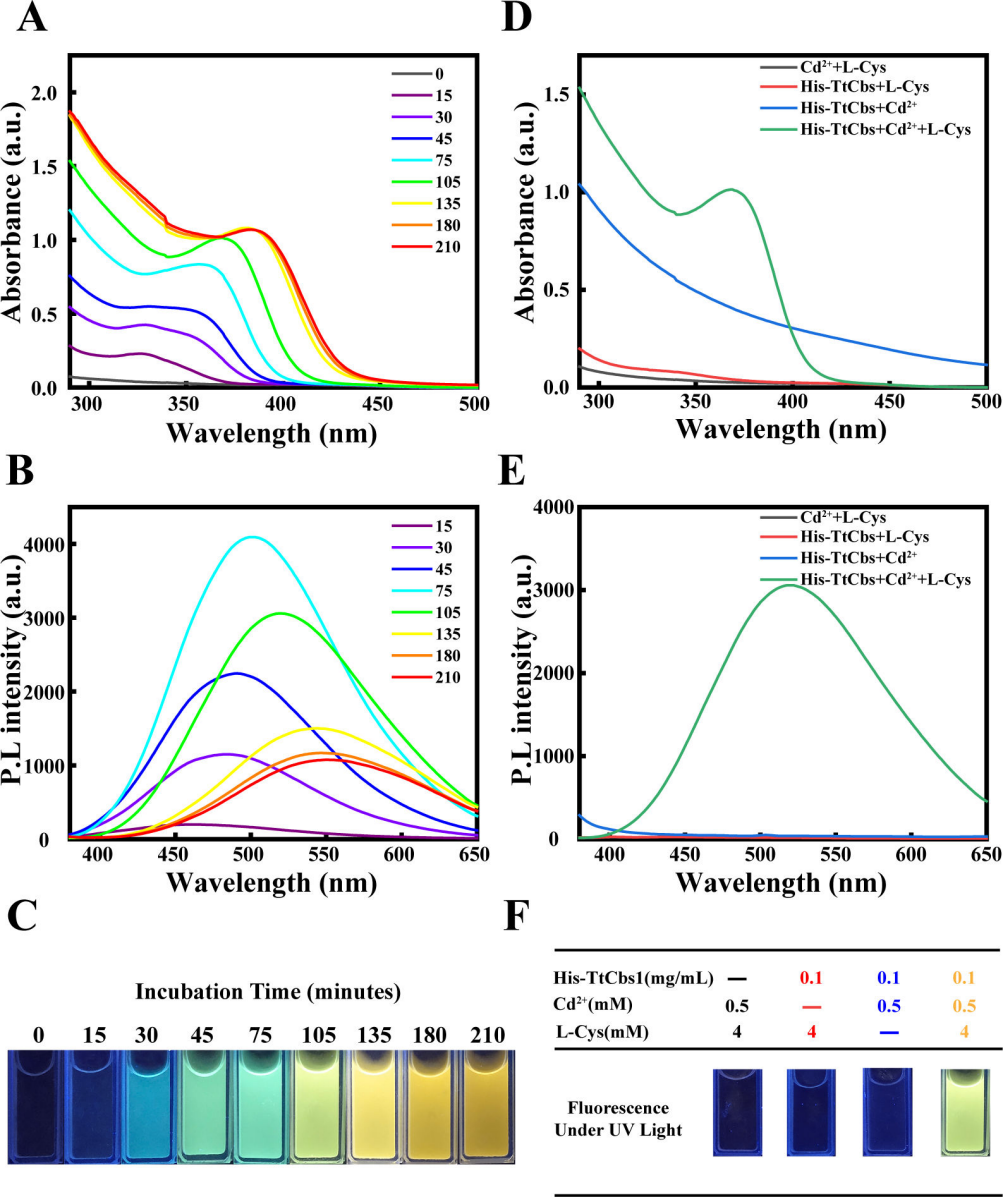
Time-dependent growth of CdS nanocrystals (**A**) UV-Vis absorption spectra. TtCbs1 (0.1 mg/mL) was incubated with 4 mM cysteine (4 mM) and cadmium chloride (0.5 mM) for various time intervals (0, 15, 30, 45, 75, 105, 135, 180, and 210 min). UV-Vis absorption spectra were recorded to monitor changes in absorbance maxima over time. A redshift in absorbance maxima was observed, indicating an increase in the size of CdS nanocrystals. (**B**) Fluorescence spectra. Corresponding fluorescence spectra were recorded with excitation at 350 nm. Fluorescence maxima exhibited a redshift at selected time intervals, consistent with the UV-Vis data. (**C**) Photoluminescent photographs. Photographs of the solutions under UV light were taken to visualize the color transition. A color transition from blue to yellow was observed, indicating an increase in the average size of CdS nanocrystals with incubation time. (**D**) UV-Vis absorption spectra of control solutions. Control solutions lacking either TtCbs1 (0.1 mg/mL), cysteine (4 mM), or cadmium chloride (0.5 mM) were incubated for 30 min. No significant absorption peaks were observed, indicating the necessity of all components for CdS nanocrystal formation. (**E**) Fluorescence spectra of control solutions. Corresponding fluorescence spectra were recorded for the control solutions. No detectable fluorescence was observed, confirming the absence of CdS nanocrystals. (**F**) Photoluminescent images of control solutions. Photoluminescent images were taken under UV light. No visible photoluminescence was observed, further confirming the necessity of all components for CdS nanocrystal formation.

To analyze His-TtCbs1 enzymatic activity for the synthesis of CdS QDs *in vitro*, the His-TtCbs1 was expressed and purified using nickel affinity chromatography and gel filtration chromatography (Fig. S1). In a reaction mixture containing Tris-HCl (50 mM, pH 8.5), His-TtCbs1 (0.1 mg/mL), cysteine (4 mM), and Cd^2+^ (0.5 mM), UV-Vis absorption spectra and fluorescence emission spectra were analyzed. After a 0.5 h reaction period, a significant UV absorption peak at 360 nm and a fluorescence emission peak at 485 nm were observed (Fig. 2A and B). As the reaction time increased, both the UV absorption peak and fluorescence emission peak exhibited a red shift, indicating an increase in the size of the generated CdS QDs. Under UV irradiation, the QDs exhibited photoluminescence, demonstrating typical quantum size effects. The color transition from blue to orange-yellow indicated an increase in particle size (22, 23) (Fig. 2C). Notably, when any one of His-TtCbs1, cysteine, or Cd^2+^ was omitted from the reaction mixture, no UV absorption peaks, fluorescence emission peak, or visible photoluminescence were observed (Fig. 2D through F).

### The synthesized CdS QDs are monodisperse and crystalline

**Fig 3:**
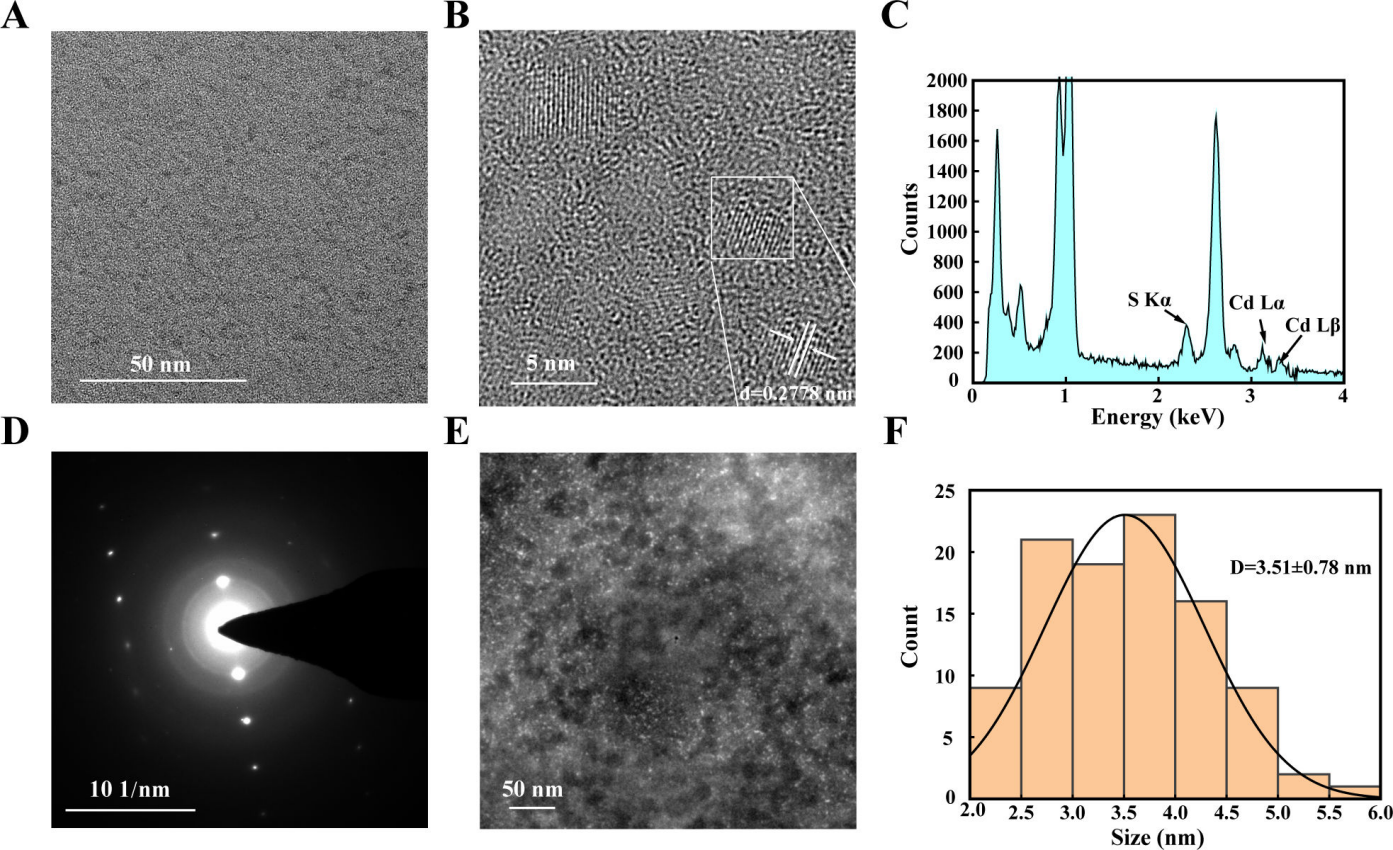
Comprehensive characterization of CdS nanocrystals. (**A**) Morphology of CdS nanocrystals. Transmission electron microscopy (TEM) was used to observe the morphology of the biosynthesized CdS nanocrystals. A representative TEM image shows numerous dispersed CdS nanocrystals. (**B**) High-resolution TEM (HRTEM) observation. HRTEM images reveal clear lattice fringes characteristic of crystalline CdS. (**C**) Energy-dispersive X-ray spectroscopy (XEDS) analysis. XEDS analysis indicates the presence of cadmium (Cd) and sulfur (S) in the synthesized quantum dots. (**D**) Selected area electron diffraction (SAED) patterns. SAED patterns indicate the crystalline nature of the CdS nanocrystals, with distinct diffraction rings corresponding to the crystal planes of CdS. (**E**) High-angle annular dark-field (HAADF) imaging. HAADF imaging provides a clear visualization of the distribution of Cd within the nanocrystals. (**F**) Size distribution analysis. ImageJ software was used to analyze the size distribution of the nanocrystals. Size distribution analysis reveals an average particle size of 3.51 nm.

TEM observations revealed that the reaction system produced uniformly distributed QDs (Fig. 3A), exhibiting typical lattice fringes (Fig. 3B). XEDS analysis confirmed the presence of sulfur and cadmium elements in the generated particles (Fig. 3C). SAED patterns indicated the formation of single-crystal CdS QDs (Fig. 3D). HAADF imaging demonstrated the even distribution of QDs (Fig. 3E), with an average particle size of 3.51 nm (Fig. 3F).

**Fig 4:**
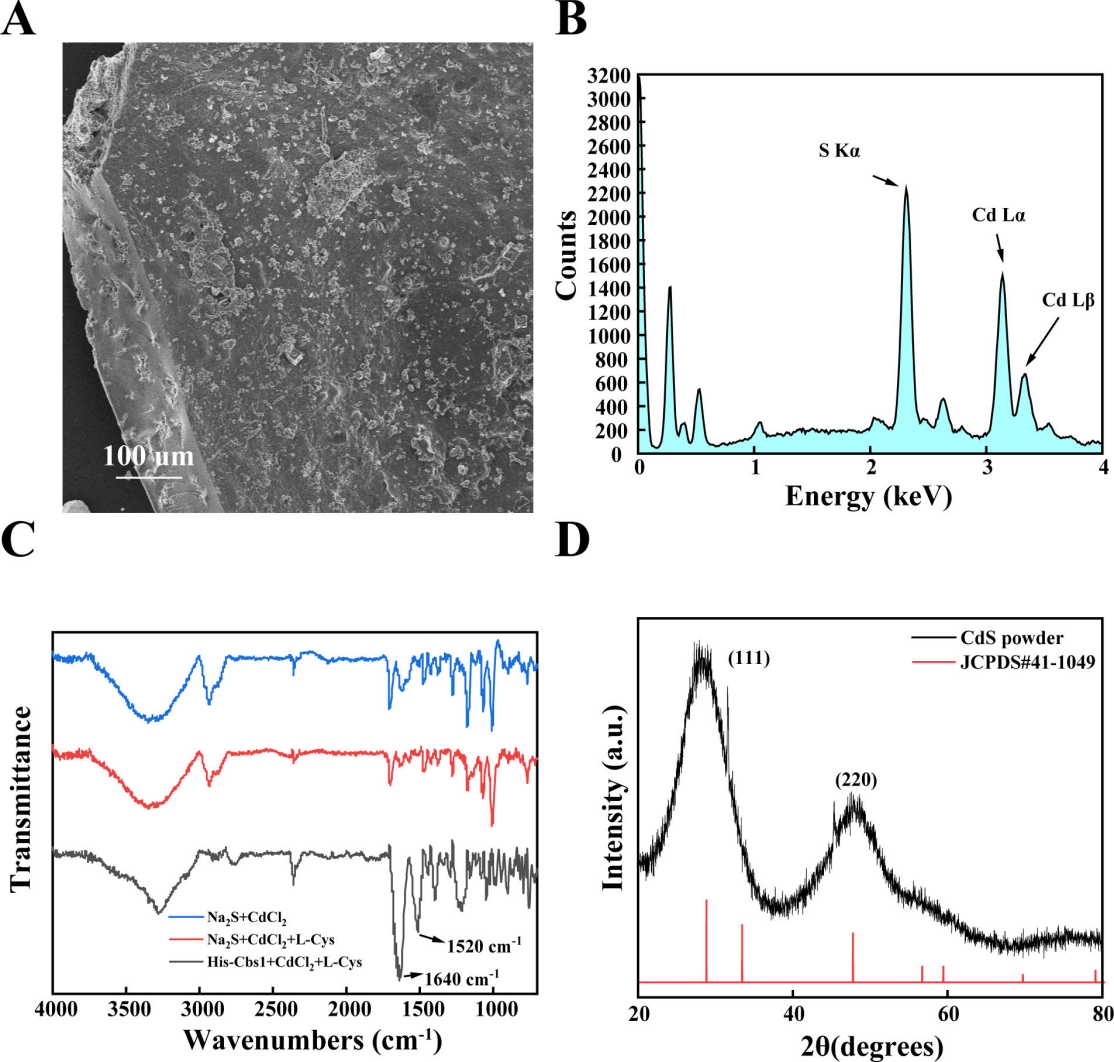
Characterization of bulk CdS. (**A**) Morphology of Bulk CdS. Scanning electron microscopy (SEM) images illustrate the formation of larger, aggregated particles compared to the nanocrystals. (**B**) Energy-dispersive X-ray (EDX) analysis. EDX analysis indicates the presence of cadmium and sulfur elements in the CdS QDs. (**C**) Fourier-transform infrared (FTIR) analysis. FTIR spectra reveal peaks at 1520 cm^−1^ (amide II) and 1640 cm^−1^ (amide I). The presence of these peaks indicates an interaction between the synthesized CdS and the His-TtCbs1 protein. (**D**) The X-ray diffraction (XRD) pattern. XRD pattern indicates the formation of wurtzite CdS, with distinct peaks corresponding to the crystal planes of the wurtzite structure.

When the reaction time was extended to 48 h, SEM observations and EDS analysis confirmed the formation of bulk CdS (Fig. 4A and B). FTIR revealed peaks at 1,520 cm⁻¹ and 1,640 cm⁻¹, characteristic of amide I and amide II bonds, respectively. This indicates an interaction between the generated CdS and the protein (41) (Fig. 4C). The XRD pattern exhibited diffraction peaks at 28.18° and 47.96°, corresponding to the (111) and (220) crystal planes of CdS (JCPDS no. 21-0829) (Fig. 4D).

### Cysteine and glutathione enhance the stability of CdS QDs

**Fig 5:**
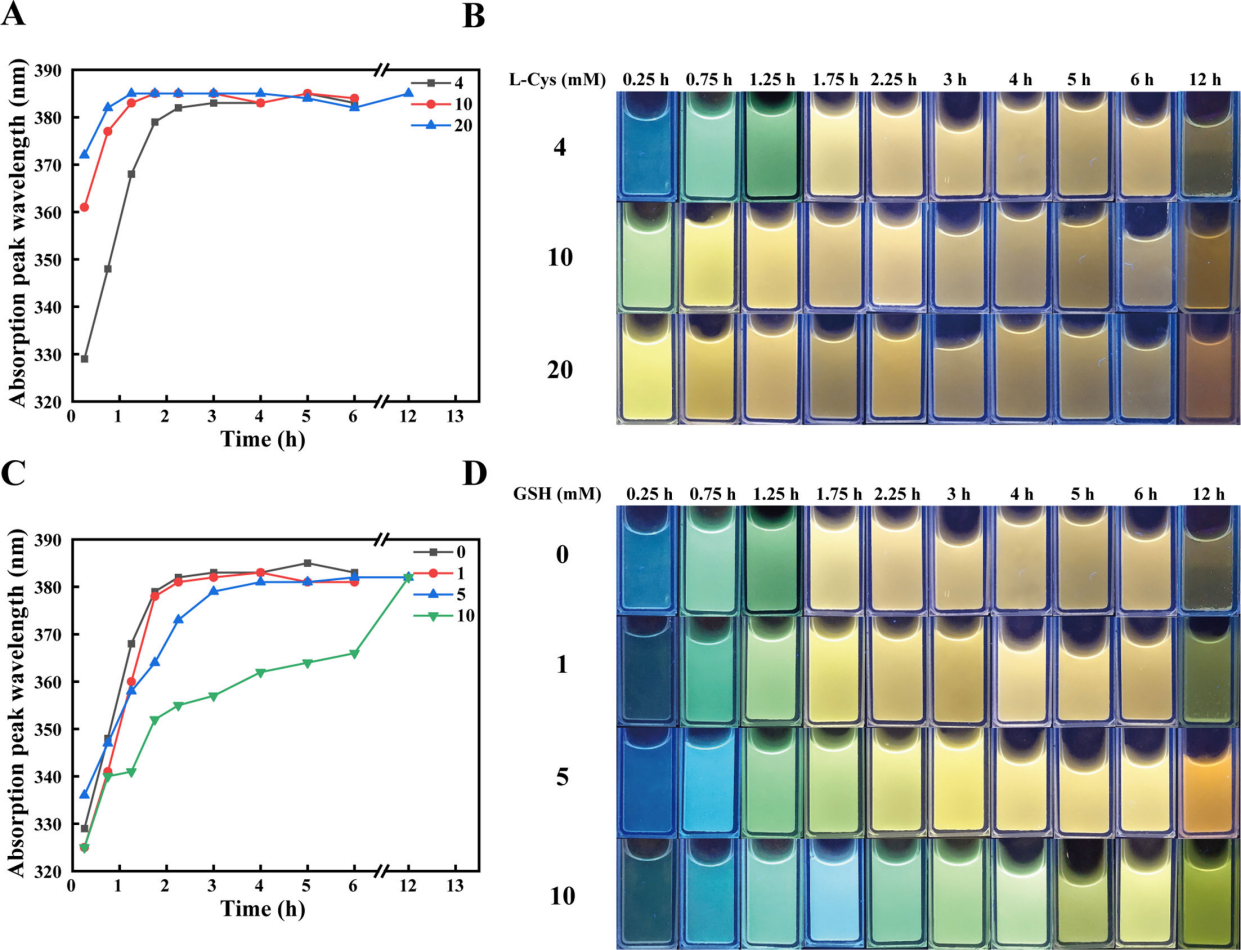
Influence of cysteine and glutathione on CdS nanocrystals size. (**A**) UV absorption peaks under different cysteine concentration. CdS nanocrystals were synthesized in the presence of varying concentrations of cysteine (4, 10, and 20 mM). UV-Vis absorption spectra were recorded over time to monitor changes in absorbance maxima. (**B**) Corresponding photographs of the solutions (**A**) under UV light. Corresponding photographs of the solutions under UV light were taken to visualize the color transition. A color transition was observed as the cysteine concentration increased. (**C**) UV absorption peaks under different glutathione concentrations. CdS nanocrystals were synthesized in the presence of varying concentrations of glutathione (0, 1, 5, and 10 mM) with a fixed 4 mM cysteine concentration. UV-Vis absorption spectra were recorded over time to monitor changes in absorbance maxima. (**D**) Corresponding photographs of the solutions (**C**) under UV light. A similar color transition was observed with increasing glutathione concentration.

In the process of generating QDs through a single enzyme system, thiols play a crucial role, not only facilitating the formation of QDs but also maintaining their particle size stability (32, 42). Cysteine, as a thiol compound, serves as a substrate for His-TtCbs1 to produce H_2_S. To investigate the additional role of cysteine, we added 4, 10, and 20 mM of cysteine as substrates in the reaction. After 0.25 h, UV absorption peaks at 329, 361, and 372 nm were observed, displaying a concentration-dependent increase. Photoluminescence under UV light revealed that the QDs emitted blue, green, and yellow light, respectively, indicating that the increase in cysteine concentration enhanced the generation of QDs (Fig. 5A and B) (22, 43). As the reaction time was extended, the UV absorption peaks gradually intensified, and the CdS QDs transitioned to larger particle sizes. After 12 h of reaction, the systems with 4 and 10 mM cysteine as substrates exhibited aggregation of CdS, while the system with 20 mM cysteine maintained the stability of the CdS QDs (Fig. 5B). This indicates that cysteine, in addition to acting as a substrate for H_2_S production, also functions as a capping agent, enhancing the stability of CdS QDs.

Glutathione, another thiol compound, often acts as a capping agent in the synthesis of QDs (22). Using 4 mM cysteine as the substrate, we investigated the functional role of glutathione by adding concentrations of 0, 1, 5, and 10 mM. The addition of glutathione led to a dose-dependent decrease in the UV absorption peaks. Notably, 5 and 10 mM of glutathione promoted the stability of CdS QDs even after 12 h (Fig. 5C and D). Further investigation revealed that the addition of glutathione did not affect the rate of cysteine consumption (Fig. S2). Additionally, glutathione did not serve as a substrate in the enzyme-catalyzed generation of QDs (Fig. S3). Therefore, the decrease in ultraviolet absorbance peak upon the addition of glutathione can be attributed to its role as a capping agent.

### CdS QDs decolorize methyl orange

**Fig 6:**
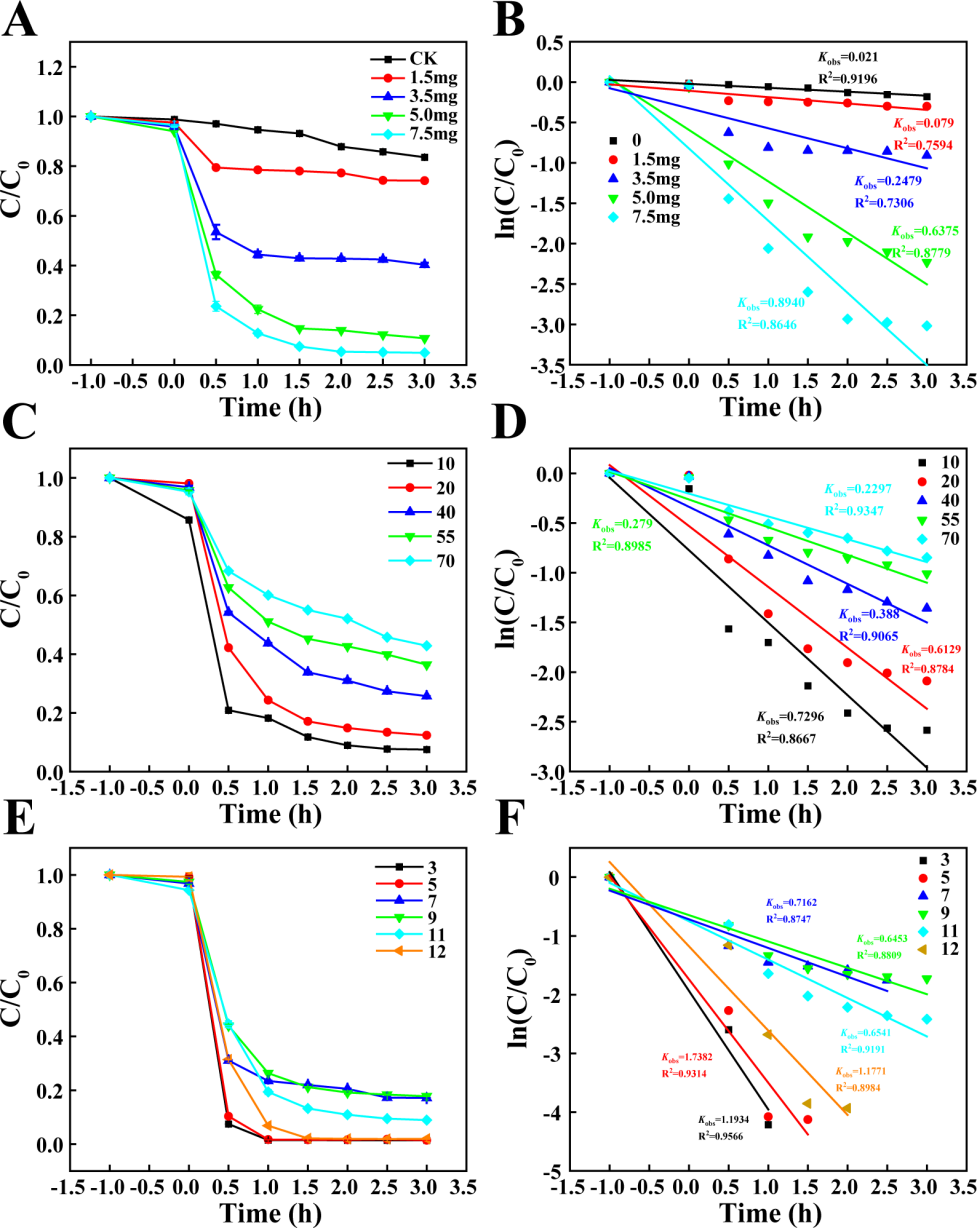
Analysis of methyl orange decolorization by CdS QDs under UV light. (**A**) Effect of CdS concentration on methyl orange decolorization. Decolorization curves of 20 mg/L methyl orange using different amounts of CdS (0, 1.5, 3.5, 5, and 7.5 mg) were evaluated. Higher concentrations of CdS led to more efficient decolorization of methyl orange. (**B**) Kinetics of methyl orange decolorization. The kinetic curve and apparent rate constants (*k*_app_) were determined for the data in panel (**A**). 7.5 mg of CdS exhibited the highest catalytic rate. (**C**) Effect of methyl orange concentration on decolorization. Decolorization curves of methyl orange at various concentrations (10, 20, 40, 55, and 70 mg/L) using 7.5 mg of CdS were evaluated. The decolorization rate decreased with increasing dye concentration. (**D**) Kinetics of methyl orange decolorization at different concentrations. The kinetic curve and apparent rate constants (*k*_app_) for the data in (**C**) were determined. The decolorization rate was highest at 10 mg/L, with *k*_app_ values decreasing from 0.7296 to 0.2297 min⁻^1^ as the concentration increased. (**E**) Effect of pH on methyl orange decolorization. Decolorization curves of methyl orange at different pH levels (ranging from 3.0 to 12.0) using CdS were evaluated. The decolorization rate was higher in acidic environments. (**F**) Kinetics of methyl orange decolorization at different pH levels. The kinetic curve and apparent rate constants (*k*_app_) for the data in (**E**) were determined. The decolorization rate was highest at pH 3.0, with a gradual decrease in *k*_app_ values as the pH increased.

To analyze the photocatalytic activity of biosynthesized CdS QDs, the decolorization efficiency of methyl orange was assessed. The decolorization of methyl orange by CdS QDs was concentration-dependent. Specifically, 7.5 mg of CdS QDs effectively decolorized 90% of a 20 mg/L methyl orange solution within 3 h (Fig. 6A). The catalytic kinetics curve indicated that 7.5 mg of CdS QDs exhibited a higher catalytic rate compared to other concentrations (Fig. 6B).

To elucidate the molecular characteristics of methyl orange during the photocatalytic decolorization process, changes in its UV-Vis absorption spectrum were monitored. The initial absorption peak at 464 nm significantly decreased as the photocatalytic reaction progressed, accompanied by a color change from orange to colorless (Fig. S4). Under a specific concentration of CdS QDs (7.5 mg), the decolorization efficiency of methyl orange at varying initial concentrations (10, 20, 40, 55, and 70 mg/L) was analyzed. The decolorization rate was highest at 10 mg/L, while the decolorization rate gradually decreased with increasing dye concentration. The *k*_app_ value decreased from 0.7296 min⁻¹ to 0.2297 min⁻¹ as the concentration increased (Fig. 6C and D). The color change of methyl orange indicates that the color gradually lightened over the decolorization time (Fig. S5). Furthermore, the decolorization rate was higher in acidic environments (Fig. 6E and F).

## DISCUSSION

H_2_S is a key player in cadmium detoxification across diverse organisms. In plants, H_2_S is rapidly activated in response to Cd²^+^ stress, preceding cysteine accumulation (16). This early response underscores H₂S’s role as a primary signal for initiating stress adaptation. In *P. stutzeri* 273, threonine dehydrogenase knockout reduced H_2_S production, impairing Cd removal efficiency (22). Conversely, overexpressing cysteine desulfhydrase in *E. coli* enhanced CdS mineralization (44). Our previous work showed that the cysteine synthase TtCsa1, involved in the *de novo* cysteine synthesis, is crucial for CdS biomineralization in *T. thermophila* (32). In this study, we observed that significant upregulation of *TtCBS1* under Cd stress peaked at 25 µM Cd. Further increases in Cd concentration (>50 µM) led to a decline in *TtCBS1* expression, indicating oxidative stress-mediated metabolic inhibition. However, supplementing with cysteine further upregulated *TtCBS1* expression, suggesting that cysteine may effectively alleviate cadmium toxicity (Fig. 1).

Recombinant CSE single-enzyme enzyme system utilizes cysteine to generate H₂S. At elevated pH values, H₂S dissociates into HS⁻, providing an essential sulfur source for the synthesis of CdS QDs. Subsequently, HS⁻ reacts with Cd^2+^ to form CdS QDs (45). The reaction time and the choice of capping agents significantly influence particle size of CdS (23, 46). In this study, we observed that the CdS QDs catalyzed by His-TtCbs1 exhibited a redshift in both UV absorption and fluorescence emission peaks as the reaction time increased. This indicates continuous enlargement of the particle size. Under UV light, the photoluminescence transitioned from a blue color, characteristic of smaller particles, to a yellow color associated with larger particles (Fig. 2). Both cysteine and glutathione served as effective capping agents, stabilizing CdS in its QD form (Fig. 4). The single-enzyme system utilizing His-TtCbs1 for CdS QDs synthesis demonstrates significant controllability over particle size through reaction time and capping agent selection. This system’s ability to produce stable CdS QDs with tunable properties highlights its potential for green synthesis applications, offering a sustainable alternative to traditional chemical methods.

CdS is a widely studied semiconductor material known for its unique optoelectronic properties, making it a promising candidate for photocatalytic applications (47, 48). The photocatalytic performance of biosynthesized CdS QDs was assessed by degrading methyl orange under UV light. The CdS QDs achieved 90% decolorization of a 20 mg/L methyl orange solution within 3 h. The decolorization rate decreased with increasing dye concentration. Higher concentrations of methyl orange obstructed light penetration, reducing the effective surface area of CdS QDs exposed to UV light and thereby diminishing catalytic efficiency (38). The observed concentration-dependent efficiency highlights the importance of optimizing reaction conditions for practical applications.

The decolorization rates of CdS QDs were notably higher in both acidic and alkaline conditions compared to neutral pH. In acidic conditions, the CdS QD surface becomes protonated, enhancing the adsorption of anionic methyl orange dye molecules. This improved adsorption facilitates closer proximity between the dye and the photocatalyst, thereby increasing the likelihood of reactive species interaction. In alkaline media, the increased accumulation of hydroxide ions (OH⁻) on the surface of CdS. The presence of OH⁻ ions at the adsorption sites enhances the generation of ·HO, which are highly reactive and promote the photocatalytic process (49). Under UV light irradiation, the electrons in CdS QDs are excited and transition to the conduction band, generating photoelectrons (e^−^). These photogenerated electrons reduce O_2_ to form superoxide anions (·O_2_^−^). The holes (h^+^) located in the valence band of CdS react with hydroxyl ions (OH^−^) to generate hydroxyl radicals (·OH), which are crucial for the decolorization of organic dyes during the photochemical reaction (Fig. S6) (50). The generation of reactive species like hydroxyl radicals and superoxide anions is highly dependent on the pH conditions, which in turn impacts the overall photocatalytic activity. Understanding these pH-dependent mechanisms is essential for optimizing the photocatalytic performance of CdS QDs in practical applications.

Traditionally, the decolorization of methyl orange relies heavily on nanomaterials synthesized through chemical methods, which often require stringent conditions such as high temperatures, pressures, and the use of organic reagents. The performance of CdS is compared with previously reported photocatalysts for the removal of methyl orange in Table S1. The results indicate that the CdS synthesized through enzyme-mediated processes demonstrates comparable catalytic efficiency to the catalysts reported in the literature, highlighting the superior photocatalytic decolorization capabilities of the catalyst developed in this study (Table S1). Our study utilized the His-TtCbs1 single enzyme system to synthesize CdS QDs at room temperature, offering a green and sustainable alternative to traditional chemical methods. The biosynthesized CdS QDs achieved a 91% decolorization rate of methyl orange within 120 min, comparable to chemically synthesized materials. However, the CdS QDs synthesized in this study did not effectively degrade dye molecules under visible light, limiting their applicability in broader environmental scenarios. While the current CdS QDs are limited in visible light activity, future research into material combinations and interface engineering holds promise for enhancing their applicability in broader environmental remediation scenarios (51, 52).

### Conclusion

In summary, this research confirms the involvement of *TtCBS1* in the response to cadmium stress in *T. thermophila*. The single-enzyme system facilitated the generation of monodisperse CdS QDs *in vitro*, with cysteine acting both as a substrate and a capping agent. This progress proved effective in degrading methyl orange under UV light. This study further expands the scope of single-enzyme systems as a novel and effective method for synthesizing QDs, presenting promising prospects for their application in the field of photocatalytic dye decolorization.

## Data Availability

The additional data supporting the work are available from the corresponding author upon request.

## SUPPLEMENTAL MATERIAL

Graphical abstract aem.01255-25-s0001.tif Visual diagram of the study.

Supplemental material aem.01255-25-s0002.pdf Figures S1 to S6; Table S1.

ASM does not own the copyrights to Supplemental Material that may be linked to, or accessed through, an article. The authors have granted ASM a non-exclusive, world-wide license to publish the Supplemental Material files. Please contact the corresponding author directly for reuse.
