# Supplementary figures and images for: Cystathionine β-synthase TtCbs1 from Tetrahymena thermophila catalyzes the synthesis of CdS quantum dots for methyl orange decolorization
Source: Appl Environ Microbiol. 2025 Sep 24;91(10):e01255-25. doi: 10.1128/aem.01255-25 (PMC12542638; doi:10.1128/aem.01255-25)

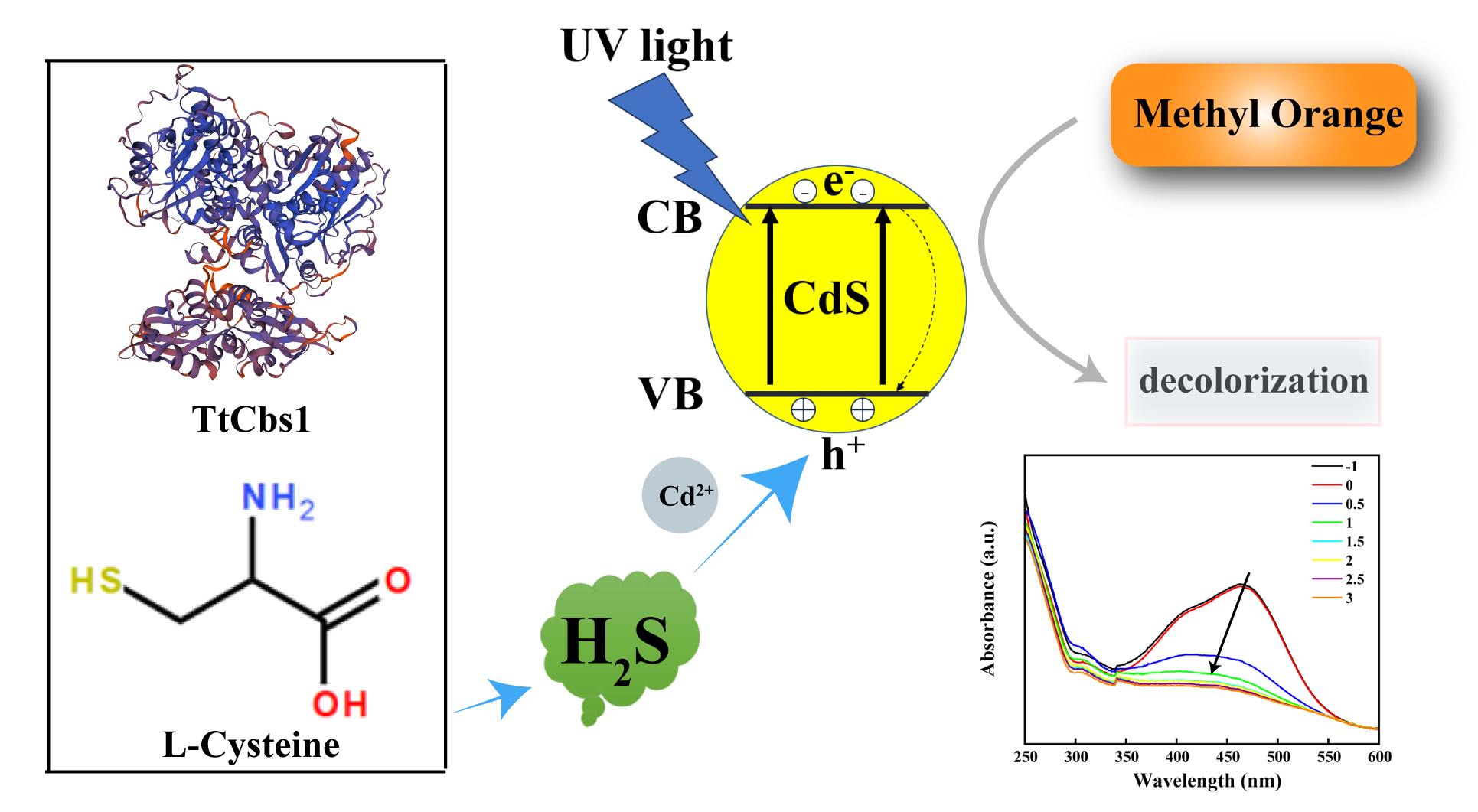

Supplement: Graphical abstract — Visual diagram of the study. [file aem.01255-25-s0001.tif]
