# Supplementary material for: Cystathionine β-synthase TtCbs1 from Tetrahymena thermophila catalyzes the synthesis of CdS quantum dots for methyl orange decolorization
Source: Appl Environ Microbiol. 2025 Sep 24;91(10):e01255-25. doi: 10.1128/aem.01255-25 (PMC12542638; doi:10.1128/aem.01255-25)
Supplement: Supplemental material — Figures S1 to S6; Table S1. [file aem.01255-25-s0002.pdf]

Cystathionine β-Synthase TtCbs1 from *Tetrahymena thermophila* Catalyzes the Synthesis of CdS Quantum Dots for Methyl Orange Decolorization

Wenliang Lei[a], Juan Liu[a], Jing Xu[a,b], Wei Wang[a,c*]

a Key Laboratory of Chemical Biology and Molecular Engineering of Ministry of Education, Institute of Biotechnology, Shanxi University, Taiyuan 030006, China.

b School of Life Science, Shanxi University, Taiyuan 030006, China.

c Shanxi Key Laboratory of Biotechnology, Taiyuan 030006, China.

* Correspondence: gene@sxu.edu.cn.

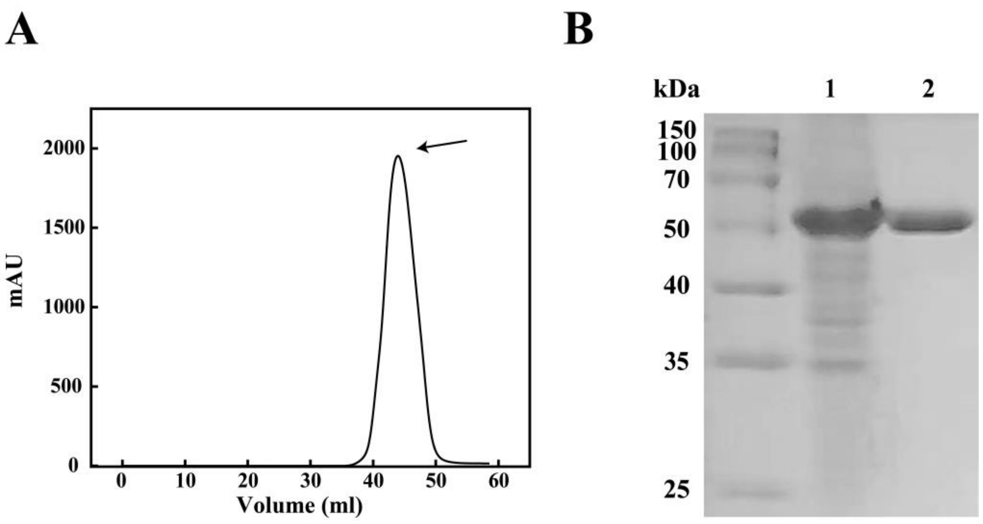

Fig. S1. Purification of TtCbs1. (A) Gel filtration chromatography profile. The purification of TtCbs1 was performed using gel filtration chromatography. The chromatography profile shows a distinct peak corresponding to the target protein, indicated by arrows. (B) SDS-PAGE analysis. SDS-PAGE analysis was conducted to confirm the purity and size of the purified His-TtCbs1. The cell lysate (Lane 1) and the purified His-TtCbs1 from gel filtration chromatography (Lane 2) were analyzed. The purified His-TtCbs1 exhibited a band corresponding to a molecular weight of 67 kDa, confirming the successful purification and expected size of the protein.

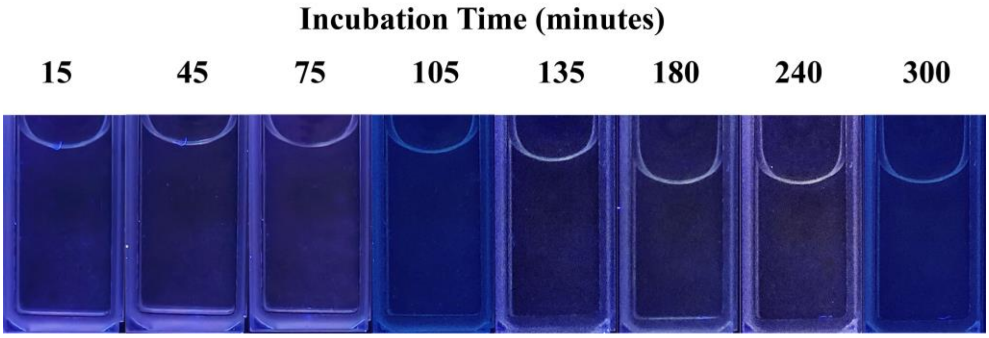

Fig. S2. CdS nanoparticles failed to form in the presence of 0.1 mg/mL His-TtCbs1, 10 mM glutathione, and 0.5 mM cadmium chloride when cysteine was omitted.

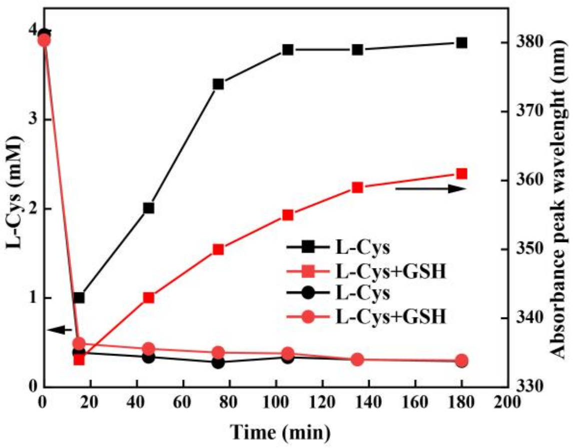

Fig. S3. Effect of glutathione on cysteine consumption in CdS nanoparticle synthesis. The addition of glutathione did not affect cysteine consumption. Under reaction conditions containing 4 mM cysteine, 0.5 mM cadmium chloride, and 0.1 mg/mL His-TtCbs1, the absorbance maximum (squares) and cysteine concentration in solution (circles) were measured over time. These measurements were taken both with (red) and without (black) the addition of 10 mM glutathione. The glutathione did not significantly alter the rate of cysteine consumption or the absorbance maximum, indicating that it does not serve as a substrate in the reaction.

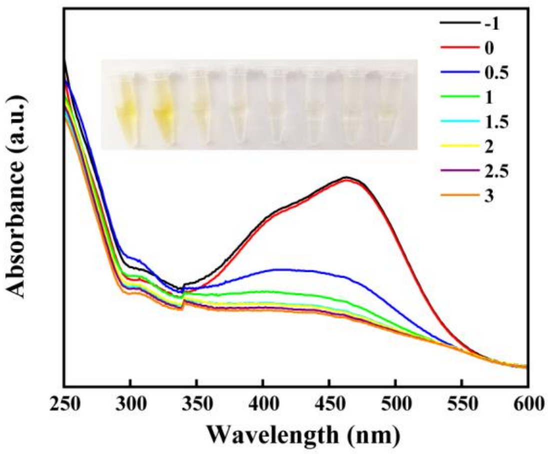

Fig. S4. UV-Vis spectra of methyl orange during photocatalytic reaction with CdS QDs. The UV-Vis absorption spectra demonstrate the degradation of methyl orange during the photocatalytic reaction with CdS QDs. Accompanying photographs show the color changes of the solutions over time (inset).

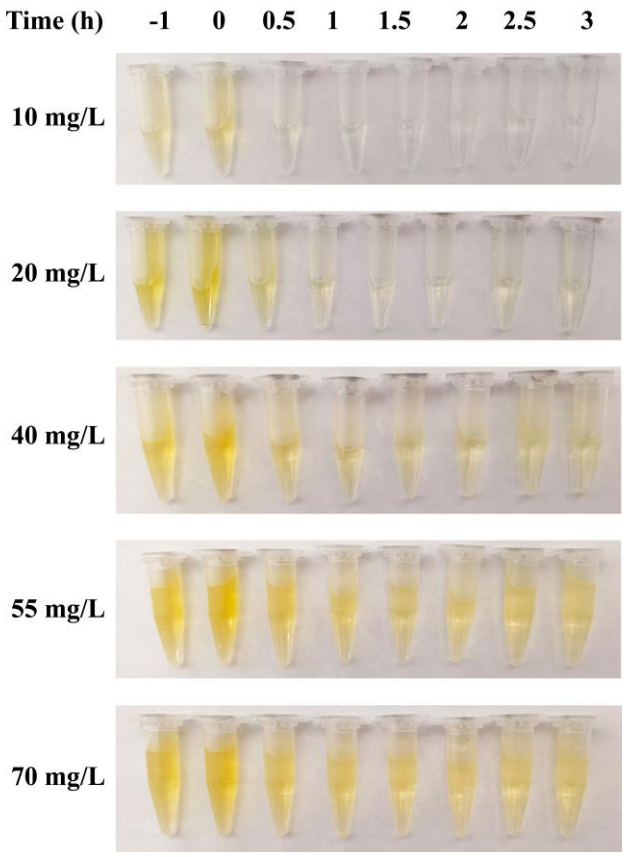

Fig. S5. Color variation of methyl orange during photodegradation mediated by CdS. The color variation of methyl orange was observed at various concentrations (10, 20, 40, 55, and 70 mg/L) during the photodegradation process mediated by QDs. The color of the solutions changed progressively as the reaction proceeded, indicating the degradation of methyl orange.

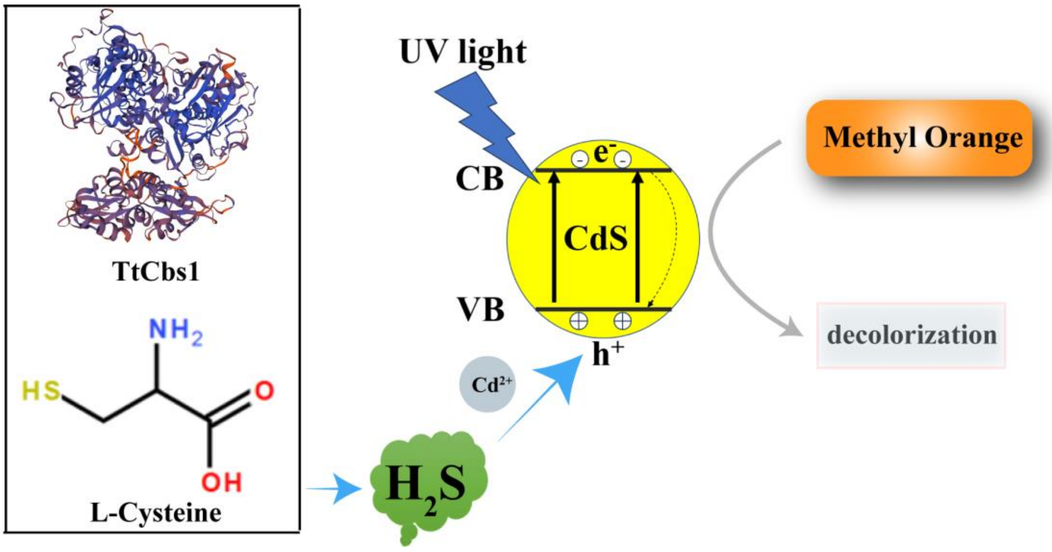

Fig. S6. Schematic diagram of the synthesis and charge transfer mechanism of CdS.

Table S1. Comparison of methyl orange removal with CdS based photocatalysts in the literature.

Catalyst	Light sources	Dyes concentration (mg/ L)	Removal efficiency	Catalyst dosage (g/L)	Reference
CdS	UV	10	120 min,81%	0.2	[1]
CdS/graphene	xenon lamp	10	120 min,94%	0.5	[2]
CdS - $BiPO_4$	UV	10	75 min,99%	1	[3]
CdS	UV	10	40 min,93%	0.2	[4]
Fe_3O_4@SiO_2/Ag/AgCl/CdS	UV	10	50 min,82%	1	[5]
CdS/ZnS	UV	10	120 min,99%	0.5	[6]
G0-MCD- TiO_2/CdS	UV	20	120 min,99%	2	[7]
CdS	UV	10	120 min, 91%	0.75	This work

Reference

[1] Hojjati-Najafabadi A, Farahbakhsh E, Gholamalian G, Feng P, Davar F, Aminabhavi T M, Vasseghian Y, Kamyab H, Rahimi H, Controllable synthesis of nanostructured flower-like cadmium sulfides for photocatalytic degradation of methyl orange under different light sources, J. Water Process Eng. 59 (2024) 105002.

[2] Chen F, Jin X, Jia D, Cao Y, Duan H, Long M, Efficient treament of organic pollutants over CdS/graphene composites photocatalysts, Appl. Surf. Sci. 504 (2020) 144422.

[3] Zhao J, Ge K, Zhao L, Zhang S, Zeng Y, Enhanced photocatalytic properties of CdS -decorated $BiPO_4$ heterogeneous semiconductor catalyst under UV-light irradiation, J. Alloys Compd. 729 (2017) 189-197.

[4] Chen F, Jia D, Cao Y, Jin X, Liu A, Facile synthesis of CdS nanorods with enhanced photocatalytic activity, Ceram. Int. 41(10) (2015) 14604-14609.

[5] Khojasteh H, Mohammadi-Aghdam S, Heydaryan K, Sharifi N, Aspoukeh P, Khanahmadzadeh S, Khezri B, Optimization of Fe_3O_4@SiO_2/Ag/AgCl/CdS nanocomposite via response surface methodology: an efficient visible-light photocatalyst for methyl orange degradation, J. Sol-Gel Sci. Technol. 111(2) (2024) 362-380.

[6] Jin X, Jiao M, Zhang X, Chen F, Zhou Z, Jia D, Visible-light-driven CdS/ZnS heterojunctions with excellent photocatalytic performance for organic dyes removal, J. Mol. Struct. 1317 (2024) 139188.

[7] Mahmood A, Park J-W, TiO_2/CdS nanocomposite stabilized on a magnetic-cored dendrimer for enhanced photocatalytic activity and reusability, J. Colloid Interface Sci. 555 (2019) 801-809.
